# Prevalence and Characterization of Food-Related Methicillin-Resistant *Staphylococcus aureus* (MRSA) in China

**DOI:** 10.3389/fmicb.2019.00304

**Published:** 2019-02-20

**Authors:** Shi Wu, Jiahui Huang, Feng Zhang, Qingping Wu, Jumei Zhang, Rui Pang, Haiyan Zeng, Xiaojuan Yang, Moutong Chen, Juan Wang, Jingsha Dai, Liang Xue, Tao Lei, Xianhu Wei

**Affiliations:** ^1^State Key Laboratory of Applied Microbiology Southern China, Guangdong Institute of Microbiology, Guangzhou, China; ^2^Guangdong Provincial Key Laboratory of Microbial Culture Collection and Application, Guangdong Open Laboratory of Applied Microbiology, Guangzhou, China; ^3^School of Bioscience and Bioengineering, South China University of Technology, Guangzhou, China; ^4^College of Food Science, South China Agricultural University, Guangzhou, China

**Keywords:** MRSA, antibiotic resistance, retail food, *spa*-typing, MLST

## Abstract

Methicillin-resistant *Staphylococcus aureus* (MRSA) is an emerging pathogen that is difficult to treat due to the multiresistance of the bacteria upon infection. From 2011 to 2016, 1581 *S. aureus* strains were isolated from 4300 samples from retail foods covering most provincial capitals in China. To determine the prevalence of food-related MRSA and its genetic background in China, antibiotic resistance, staphylococcal toxin genes, staphylococcal cassette chromosome *mec* (*SCCmec*) typing, *spa*-typing and MLST were carried out in this study. In total, 108 (7.4%) isolates were confirmed for MRSA by phenotyping (cefoxitin) and genotyping (*mecA/mecC* gene). A total of 52.8% (57/108) of the MRSA isolates belonged to clonal complex 59 (CC59) (ST59, ST338, and ST3355), which was the predominant clone in this study. These CC59 isolates carried SCC*mec* elements of type IV, V, or III and exhibited *spa* type t437, t441, t543, t163, t1785, or t3485, and half of them carried major virulence genes, such as the Panton-Valentine leucocidin (PVL) gene. The secondary clones belonged to ST9 (15.7%, 17/108) with a type of t899-SCC*mec* III and showed a broader range of antimicrobial resistance. The remaining MRSA isolates (31.5%, 34/108) were distributed in 12 different STs and 18 different *spa* types. All isolates harbored at least one of the enterotoxin genes, whereas only 4 isolates (3.70%) were positive for the toxic shock syndrome toxin *tsst* alleles. For antibiotic susceptibility testing, all isolates were resistant to more than three antibiotics, and 79.6% of the isolates were resistant to more than 10 antibiotics. Amoxycillin/clavulanic acid, ampicillin, cefoxitin, penicillin, ceftazidime, kanamycin, streptomycin, clindamycin, and telithromycin was the most common antibiotic resistance profile (55.6%, 60/108) in the study. In summary, the results of this study implied that the major food-related MRSA isolate in China was closer to community-associated MRSA, and some of the remaining isolates (ST9-t899-SCC*mec* III) were supposed to livestock-associated MRSA. In addition, most MRSA isolates showed resistance to multiple drugs and harbored staphylococcal toxin genes. Thus, the pathogenic potential of these isolates cannot be ignored. In addition, further studies are needed to elucidate the transmission routes of MRSA in relation to retail foods and to determine how to prevent the spread of MRSA.

## Introduction

Methicillin-resistant *Staphylococcus aureus* (MRSA) is a pathogen of increasing importance in hospitals as well as in the community and livestock. It can be resistant to several antibiotics and quickly disseminates worldwide. In recent years, MRSA has been attributed to an estimated 5400 extra deaths and over one million extra days of hospitalization (Kobayashi et al., 2015). *S. aureus* became MRSA because of the acquisition of the gene *mecA* or *mecC*, which encodes the low-affinity penicillin-binding protein 2a (PBP2a), which, unlike other PBPs, remains active and allows for cell wall biosynthesis at otherwise lethal β-lactam concentrations. The *mec* operon is carried by the staphylococcal cassette chromosome *mec* (SCC*mec*) and most likely originated from horizontal transfer from coagulase-negative staphylococcal species (Banerjee et al., 2008; Garcíaálvarez et al., 2011).

MRSA strains have been reported from various sources. To distinguish epidemiological groups of MRSA, it was divided into hospital-associated MRSA (HA-MRSA), community-associated MRSA (CA-MRSA) and livestock-associated MRSA (LA-MRSA) (Petinaki and Spiliopoulou, 2012). MRSA was first recognized as HA-MRSA in 1961 and then spread into the community and, later on, into healthcare facilities; in 1990, this type became recognized as CA-MRSA. LA-MRSA has always been associated with animals and is linked to a jump from animals to humans (Petinaki and Spiliopoulou, 2012). In general, the genetic backgrounds differ among different types of MRSA. In previous research, CA-MRSA strains frequently harbor SCC*mec* type IV or V and often produce potent toxins/virulence factors, such as Panton-Valentine leucocidin (PVL), arginine catabolic mobile element (ACME) and phenol-soluble modulins, while HA-MRSA strains typically possess larger-size SCC*mec* type I-III and are more resistant to other classes of antibiotics (An and Otto, 2008; Chuang and Huang, 2013). For multilocus sequence typing (MLST), sequence type 5 (ST5), ST8, ST22, ST36, and ST45 spread successfully to different regions of the world and caused substantial nosocomial disease (Deleo et al., 2010), whereas CA-MRSA showed five lineages worldwide: ST1-IV (USA400), ST8-IV (USA300), ST30-IV (Pacific/Oceania), ST59-IV and V (USA1000, Taiwan) and ST80-IV (European) (Skov, 2009). In contrast, LA-MRSA strains exhibit co-resistance to many non-β-lactam antimicrobials (e.g., antibiotics and metals), including those commonly used in animal production, and many of them belong to CC398 or CC9, as determined by MLST (Bens et al., 2006; Cui and Li, 2009; Neela et al., 2009). The SCC*mec* elements of LA-MRSA are different from those carried by other MRSA genotypes commonly found in the community and healthcare settings (Li et al., 2011). In addition, the majority of LA-MRSA isolates lack toxins, such as PVL and other enterotoxins (Hallin et al., 2011).

Nowadays, MRSA strains have been reported from various foods sources, such as poultry, pork, beef, milk and vegetables, suggesting that foods may serve as reservoirs (Wang et al., 2014; Wu et al., 2018). As is commonly known, foods have many different origins, and different types of MRSA are present in foods of different origin in different countries. In China, large-scale studies of the prevalence of *S. aureus* in food are scarce. From July 2011 to June 2016, we collected 4300 retail food samples from supermarkets, fairs and farmers’ markets that covered most of the provincial capitals of China (Supplementary Figure 1) and found 1063 (24.7%) *S. aureus*-positive samples from all sampling sites. To better understand the genetic background among food-related MRSA isolates in China, this study aimed to identify the MRSA isolates from our previous study and to characterize these MRSA isolates for their antimicrobial resistance profiles, their virulence genes and their genotypic types (SCC*mec*-, MLST, and *spa* types).

## Materials and Methods

### Bacterial Isolates

A total of 1581 *S. aureus* isolates were collected from 4300 retail food samples in 39 Chinese cities (Supplementary Figure 1), comprising 469 isolates from meat and meat products (bacon/sausage, poultry, pork, mutton and beef), 511 isolates from aquatic products (freshwater fish, shrimp and seafood), 368 isolates from quick-frozen products (frozen dumplings/steamed stuffed buns and frozen meat), 148 isolates from ready-to-eat food (cold vegetable/noodle dishes in sauce, fried rice/sushi, roast meat, sausage, and ham), 42 isolates from edible mushrooms, 30 isolates from vegetables and 13 isolates from pasteurized milk. These isolates were obtained between July 2011 and June 2016 according to the GB 4789.10-2010 food microbiological examination of *S. aureus* (National Food Safety Standards of China) and the most probable number (MPN) method (Gombas et al., 2003). They were identified by Gram stain, catalase and oxidase tests and API STAPH test strips (BioMerieux, Marcy-1’Etoile, France). Each isolate was incubated at 37°C overnight in brain heart infusion (BHI) broth. Genomic DNA was extracted using a genomic DNA extraction kit (Magen Biotech, Guangzhou, China) according to the manufacturer’s instructions. The concentration of genomic DNA was determined at 260 nm using a NanoDrop-ND-1000 UV-Vis spectrophotometer (Thermo Fisher Scientific, MA, United States).

### MRSA Confirmation

Cefoxitin disks (30 μg) were used for detecting methicillin-resistant isolates. *S. aureus* ATCC 25923 was used as a control. The *mecA/mecC* gene, which has been shown to confer methicillin resistance to *S. aureus* (MRSA), was also detected by PCR using primers as described previously (Perez-Roth et al., 2001; Stegger et al., 2012).

### Antimicrobial Susceptibility Testing

A total of 25 antimicrobial agents were tested for antimicrobial susceptibility in all MRSA isolates. Amoxycillin/clavulanic acid (AMC, 30 μg), ampicillin (AMP, 10 μg), cefepime (FEP, 10 μg), penicillin G (P, 10 U), ceftazidime (CAZ, 30 μg), amikacin (AK, 30 μg), gentamicin (CN, 10 μg), kanamycin (K, 30 μg), streptomycin (S, 25 μg), chloramphenicol (C, 30 μg), clindamycin (DA, 2 μg), erythromycin (E, 15 μg), telithromycin (TEL, 15 μg), ciprofloxacin (CIP, 5 μg), norfloxacin (NOR, 10 μg), tetracycline (TE, 30 μg), linezolid (LZD, 30 μg), trimethoprim/sulphamethoxazole 1:19 (SXT, 25 μg), rifampicin (RD, 5 μg), quinupristin/dalfopristin (QD, 15 μg), teicoplanin (TEC, 30 μg), nitrofurantoin (F, 300 μg) and fusidic acid (FD, 10 μg) were tested by the disk diffusion method using Mueller–Hinton agar and commercially available discs (Oxoid, United Kingdom). Vancomycin and daptomycin were tested by broth microdilution according to the recommendations of the Clinical and Laboratory Standards Institute (CLSI) (CLSI, 2015). *Staphylococcus aureus* ATCC25923 and *Escherichia coli* ATCC25922 were used as quality control organisms (CLSI, 2015). The isolates with linezolid resistance determined by disk diffusion were also confirmed using a microdilution test according to the CLSI method for minimum inhibitory concentrations (MICs) (CLSI, 2015). The results of the antimicrobial susceptibilities of the analyzed strains were scored according to the guidelines of the CLSI (CLSI, 2015).

### Detection of Staphylococcal Toxin Genes

All MRSA isolates were tested by PCR for the presence of 20 genes coding for staphylococcal enterotoxins (*sea, seb, sec, sed, see, seg, seh, sei, sej, sek, sel, sem, sen, seo, sep, seq, ser*, and *seu*), and the *tsst-1* gene encoding the TSST (Varshney et al., 2009) and the *lukSF-PV* (*pvl*) genes were determined by PCR according to a previously published method (Jarraud et al., 2002). The primers and PCR conditions are presented in Supplementary Table 1. The amplicons were stained with Goldview, loaded, and electrophoresed in 1.5% agarose at 120 V for 0.5 h and visualized under a UV transilluminator gel imaging system (GE Healthcare, WI, United States). The images were saved as TIFF files for analysis.

### Molecular Typing of MRSA Isolates

All MRSA isolates were subjected to SCC*mec* typing, *spa*-typing and MLST. The SCC*mec* typing method was performed on the isolates by multiplex PCR as previously described (Zhang et al., 2005). The *S. aureus* protein A (spa) repeat region was amplified according to a published protocol (Shopsin et al., 1999). The MLST scheme used to characterize MRSA isolates is based on the sequence analysis of the following seven housekeeping genes: *arcC, aroE, glpF, gmk, pta, tpi*, and *yqil* (Enright et al., 2013).

The DNA fragments were purified using a PCR purification kit (Qiagen, Genman) and sequenced in each direction with Big Dye fluorescent terminators on an ABI 3730XL sequencer (Applied Biosystems). The *spa* types were randomly assigned using the SpaServer website^1^. For each MLST locus, an allele number was given to each distinct sequence variant, and a distinct ST number was attributed to each distinct combination of alleles at the seven genes. STs were determined using the *S. aureus* MLST database^2^. Clonal complex (CC) analysis was performed in eBURST v.3 as previously described (Feil et al., 2004). The minimum spanning tree (MST) was constructed with Bionumerics 7.6 software (Applied Maths, Sint-Martens-Latem, Belgium).

## Results

### Prevalence of MRSA in Food

Overall, of the 1581 *S. aureus* isolates from retail food in China, 108 (6.83%) isolates from 89 positive samples (2.1%, 89/4300) were confirmed as MRSA, which exhibited cefoxitin resistance, 99.1% of isolates (107/108) were positive for *mecA* genes, and none were positive for the *mecC* gene. The distribution of MRSA among different sampling sites is shown in Supplementary Figure 2. In total, 29 of the 39 sampling cities (74.4%) had MRSA-positive samples, including 22 of the 24 southern cities and 7 of 15 the northern cities. Based on these results, there were 67 (2.4%) and 22 MRSA-positive samples (1.5%) in south China and north China, respectively. The most severe contamination level among the 29 cities was observed in Lasa (8%, 8/100), followed by Fuzhou (7%, 7/100), Nanchang (6%, 6/100), Shantou (5%, 5/100), Zhanjiang (4%, 4/100), Haikou (4%, 4/100), Nanning (4%, 4/100), Chengdu (4%, 4/100), Heyuan (3%, 3/100), Macao (3%, 3/100), Hangzhou (3%, 3/100), Xining (3%, 3/100), Huhehaote (3%, 3/100), Shijiazhuang (3%, 3/100), Shenzhen (2%, 2/100), Shanghai (2%, 2/100), Wuhan (2%, 2/100), Changchun (2%, 2/100), Zhengzhou (2%, 2/100), and Guangzhou (1.6%, 8/500); other cities were represented by one positive sample (Table 1). The analyzed food products were classified into seven categories, and the values of MRSA contamination in each sample were determined (Table 2). Among the analyzed categories, MRSA was detected in 4.8% (29/604) of raw meat, 3.0% (26/860) of aquatic products, 2.7% (16/601) of quick-frozen food, 1.1% (9/859) of ready-to-eat food, 1.0% (4/419) of vegetables and 0.7% (5/699) of edible mushrooms, whereas pasteurized milk was free of MRSA isolates. Of the 108 MRSA isolates, 34 (7.25%) of the 469 isolates were from raw meat, 31 (6.07%) of the 511 isolates were from aquatic products, 20 (5.43%) of the 368 isolates were from quick-frozen products, 11 (7.43%) of the 148 isolates were from ready-to-eat food, 7 (16.67%) of the 42 isolates were from edible mushrooms, and 5 (16.67%) of the 30 isolates were from vegetables, whereas pasteurized milk was free of MRSA isolates (Table 2).

**Table 1:** Prevalence of methicillin-resistant *Staphylococcus aureus* at different sampling sites.

Sampling site		Sampling time (year.month)	No. of samples	No. (%) of MRSA-positive samples
City	Province			
Guangzhou	Guangdong	2013.02-04/2013.09-10	500	8(1.6)
Shenzhen	Guangdong	2011.12/2013.05	100	2(2.0)
Shaoguan	Guangdong	2012.03/2013.05	100	1(1.0)
Zhanjiang	Guangdong	2012.01/2013.06	100	4(4.0)
Shantou	Guangdong	2012.02/2013.05	100	5(5.0)
Heyuan	Guangdong	2012.03/2013.06	100	3(3.0)
Haikou	Hainan	2012.05/2013.12	100	4(4.0)
Sanya	Hainan	2012.05/2014.01	100	1(1.0)
Beihai	Guangxi	2012.06/2013.12	100	1(1.0)
Nanning	Guangxi	2012.07/2013.11	100	4(4.0)
Fuzhou	Fujian	2012.07/2013.11	100	7(7.0)
Xiamen	Fujian	2012.08/2013.12	100	1(1.0)
Macao^∗^	–	2015.12/2016.05	100	3(3.0)
Hongkong^∗^	–	2015.12/2016.05	100	0(0.0)
Shanghai^∗^	–	2012.09/2014.04	100	2(2.0)
Hefei	Anhui	2012.09/2014.03	100	0(0.0)
Nanchang	Jiangxi	2012.09/2014.03	100	6(6.0)
Wuhan	Hubei	2012.10/2014.04	100	2(2.0)
Chengdu	Sichuan	2012.10/2014.03	100	4(4.0)
Kunming	Yunnan	2012.11/2014.05	100	3(3.0)
Changsha	Hunan	2015.06/2016.01	100	1(1.0)
Hangzhou	Zhengjiang	2015.07/2016.02	100	3(3.0)
Guiyang	Guizhou	2015.07/2016.01	100	1(1.0)
Nanjing	Jiangsu	2015.09/2016.03	100	1(1.0)
**South China**			**2800**	**67(2.4)**
Lanzhou	Gansu	2012.11/2013.08	100	0(0.0)
Haerbin	Heilongjiang	2012.11/2013.07	100	0(0.0)
Xi’an	Shaanxi	2012.12/2013.07	100	0(0.0)
Taiyuan	Shanxi	2012.12/2013.08	100	0(0.0)
Beijing^∗^	–	2012.12/2013.08	100	0(0.0)
Jinan	Shandong	2012.12/2013.08	100	1(1.0)
Changchun	Jilin	2015.08/2016.05	100	2(2.0)
Xining	Qinghai	2015.08/2016.05	100	3(3.0)
Yinchuan	Ningxia	2015.08/2016.04	100	0(0.0)
Huhehaote	Neimenggu	2015.08/2016.05	100	3(3.0)
Shenyang	Liaoning	2015.09/2016.03	100	0(0.0)
Shijiazhuang	Hebei	2015.10/2016.03	100	3(3.0)
Zhengzhou	Henan	2015.10/2016.04	100	2(2.0)
Lasa	Tibet	2015.11/2016.05	100	8(8.0)
Wulumuqi	Xinjiang	2015.11/2016.06	100	0(0.0)
**North China**			**1500**	**22(1.5)**

*^∗^These cities are direct-controlled municipalities.*

**Table 2:** Prevalence of methicillin-resistant *Staphylococcus aureus* in different retail foods.

Types of product	No. of samples	No. (%) of MRSA samples	No. of *S. aureus* isolates	No. (%) of MRSA isolates
Raw meat	604	29(4.8)	469	34(7.25)
Aquatic products	860	26(3.0)	511	31(6.07)
Ready-to-eat food	859	9(1.1)	148	11(7.43)
Quick-frozen food^a^	601	16(2.7)	368	20(5.43)
Edible mushrooms	699	5(0.7)	42	7(16.67)
Vegetables	419	4(1.0)	30	5(16.67)
Pasteurized milk	258	0(0)	13	0(0.00)
Total	4300	89(2.1)	1581	108(6.83)

*^*a*^All quick-frozen foods were stored at -10°C before being sold*.

### Antibiotic Resistance Profiles of MRSA Isolates

The antibiotic susceptibility results of 108 MRSA isolates are shown in Table 3. All MRSA isolates were resistant to more than three antibiotics, including 33.3% of the isolates that were resistant to 4–10 antibiotics, 46.3% of the isolates that were resistant to 11–15 antibiotics and 20.4% of the isolates that were resistant to 16–26 antibiotics. The isolates were susceptible to linezolid, vancomycin and daptomycin, and the frequencies of resistance to individual agents were 100% for ampicillin and penicillin G, followed by 97.2% for ceftazidime, 87.0% for amoxicillin/clavulanic acid, 83.3% for erythromycin, 79.6% for clindamycin, 75.9% for kanamycin, 74.1% for telithromycin, 67.6% for streptomycin, 65.7% for cefepime and tetracycline, 38.0% for chloramphenicol, 27.8% for gentamycin, ciprofloxacin and fusidic acid, 25.9% for norfloxacin, 22.2% for amikacin, 13.95% for quinupristin/dalfopristin, 13.0% for trimethoprim/sulphamethoxazole 1:19, 7.4% for rifampicin, 2.8% for nitrofurantoin and 0.9% for teicoplanin. In different types of food products, the resistance to most antibiotics were equally distributed. Norfloxacin, clindamycin, quinupristin/dalfopristin, gentamycin, and trimethoprim/sulphamethoxazole 1:19 resistance was rare in MRSA isolated from non-animal sources (i.e., edible mushrooms and vegetables). It is worth noting that amikacin was the most commonly observed resistance antibiotic, whereas quinupristin/dalfopristin, gentamycin, ciprofloxacin and norfloxacin resistance were observed in low frequencies in aquatic product-related MRSA isolates compared to other types of food isolates (Supplementary Figure 2). However, AMC-AMP-FOX-P-CAZ-K-S-DA-TEL was the most common antibiotic resistance profile (55.6%, 60/108) in this study. All selected antibiotics were grouped into 15 classes of agents. Five MRSA isolates exhibited resistance to only β-lactam antibiotics, whereas 97 isolates exhibited a multidrug resistance phenotype, with resistance to ≥3 classes of antimicrobial agents.

**Table 3:** Results of antimicrobial susceptibility tests of 108 methicillin-resistant *Staphylococcus aureus* isolates obtained from retail food in China.

Antimicrobial group	Antibiotics	No. of resistant strains (%)	No. of intermediate-resistance strains (%)	No. of susceptible strains (%)
β-Lactams	Amoxicillin/clavulanic acid (AMC)	94 (87.0)	–	14(13.0)
	Ampicillin (AMP)	108(100.0)	–	0(0.0)
	Cefepime (FEP)	71(65.7)	28(25.9)	9(8.3)
	Cefoxitin (FOX)	108(100.0)	–	0(0.0)
	Penicillin G (P)	108(100.0)	–	0(0.0)
	Ceftazidime (CAZ)	105(97.2)	0	3(2.8)
Aminoglycosides	Amikacin (AK)	24(22.2)	41(38.0)	43(39.8)
	Gentamicin (CN)	30(27.8)	1(0.9)	77(71.3)
	Kanamycin (K)	82(75.9)	8(7.4)	18(16.7)
	Streptomycin (S)	73(67.6)	27(25.0)	8(7.4)
Phenicols	Chloramphenicol (C)	41(38.0)	32(29.6)	35(32.4)
Lincosamides	Clindamycin (DA)	86(79.6)	4(3.7)	18(16.7)
Macrolides	Erythromycin (E)	90(83.3)	3(2.8)	15(13.9)
	Telithromycin (TEL)	80(74.1)	8(7.4)	20(18.5)
Fluoroquinolones	Ciprofloxacin (CIP)	30(27.8)	14(13.0)	64(59.3)
	Norfloxacin (NOR)	28(25.9)	9(8.3)	71(65.7)
Tetracyclines	Tetracycline (TE)	71(65.7)	2(1.9)	35(32.4)
Oxazolidinones	Linezolid (LZD)	0(0.0)	–	108(100.0)
Ansamycins	Rifampicin (RD)	8(7.4)	3(2.8)	97(89.8)
Sulfonamides	Trimethoprim/sulphamethoxazole 1:19 (SXT)	14(13.0)	0(0.0)	93(86.1)
Quinolones	Quinupristin/dalfopristin (QD)	15(13.9)	13(12.0)	80(74.1)
Glycopeptides	Teicoplanin (TEC)	1(0.9)	39(36.1)	68(63.0)
	Vancomycin (VAN)	0(0.0)	0(0.0)	108(100.0)
Lipopeptides	daptomycin (DAP)	0(0.0)	–	108(100.1)
Nitrofurantoins	Nitrofurantoin (F)	3(2.8)	14(13.0)	91(84.3)
	Fusidic acid (FD)	30(27.8)	–	78(72.2)
Antimicrobial				
	0–3 Antimicrobials	0(0.0)		
	4–10 Antimicrobials	36(33.3)		
	11–15 Antimicrobials	50(46.3)		
	16–26 Antimicrobials	22(20.4)		

### Distribution of Virulence Genes

**FIGURE 1:**
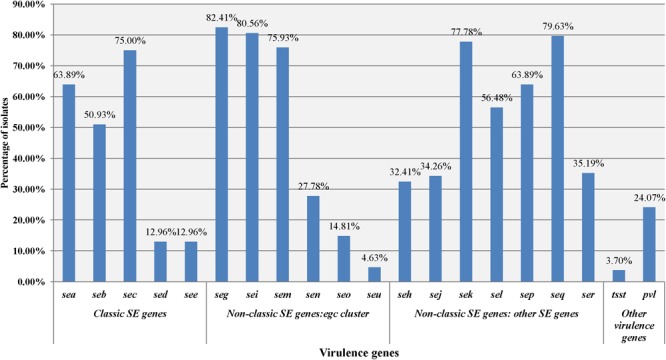
Distribution of the staphylococcal toxin gene profiles of MRSA isolates in different food types.

A total of 108 isolates of MRSA from retail food were detected for the presence of virulence genes. As shown in Figure 1, each isolate harboured at least one of the virulence genes, including 51 isolates carrying more than 10 genes. In total, 24.07% of isolates (26/108) were positive for the PVL gene *lukSF-PV*, whereas only 4 isolates (3.70%) were positive for the toxic shock syndrome toxin *tsst* alleles. Of the 18 investigated enterotoxin genes, the gene *seg* (82.41%, 88/108) was the most frequently detected, followed by the *sei* (80.56%, 86/108), *seq* (79.63%, 85/108), *sek* (77.78%, 82/108), *sem* (75.93%, 81/108), *sec* (75.00%, 79/108), *sea* (63.89%, 68/108), *sep* (63.89%, 67/108), *sel* (56.48%, 59/108), *seb* (50.93%, 54/108), *ser* (35.19%, 38/108), *sej* (34.26%, 37/108), *seh* (32.41%, 35/108), *sen* (27.78%, 30/108), *seo* (14.81%, 16/108), *see* (12.96%, 14/108), *sed* (12.96%, 14/108), and *seu* (4.63%, 5/108). In this study, 107 of 108 MRSA (99.07%) harboured one or more genes for classic SEs (*sea, seb, sec, sed*, and *see*), whereas 94 of 108 MRSA isolates (87.04%) harboured the genes of the *egc* cluster (*seg, sei, sem, sen, seo*, and *seu*). The classic SE genes showed 23.4% (195/833) of the detected genes, whereas the *egc* cluster accounted for 29.4% (245/833). Furthermore, some reported combinations of virulence genes of *S. aureus* were observed. The *sec-sel* gene combination, typical of the SaPIbov pathogenicity island, was harboured by 49.07% (53/108) of the isolates. The *sea-sek-seq* genes, which have been reported on ΦSa3ms and ΦSa3mw, were associated in 43.52% (47/108) of the isolates. In addition, *seb-sek-seq*was observed on SaIP3, and*sed-sej-ser*was observed on pIB485, which also showed 47.22% (51/108) and 11.11% (12/108) of the isolates, respectively.

### Molecular Types of MRSA Isolates

The molecular typing results are summarized in Table 4. For MLST, the STs of one strain (Sta2529-1) could not be determined. A total of 107 food-related MRSA isolates were assigned to 16 different STs, including ST1, ST5, ST6, ST7, ST9, ST10, ST45, ST59, ST88, ST188, ST338, ST398, ST630, ST943, ST3304, and ST3355. ST59 was the predominant clone and was observed in 47.7% of MRSA isolates (51/107). The remaining strains belonged to ST9 (15.9%, 17/107), ST1 (8.4%, 9/107), ST398 (6.5%, 7/107), ST7 (4.7%, 5/107), ST338 (4.7%, 5/107), ST630 (2.8%, 3/107), ST188 (1.9%, 2/107), and other STs. Based on eBURST analysis, three CCs were identified, including CC59 (ST59, ST338 and ST3355), CC7 (ST7, ST943), and CC1 (ST1, ST3304). *spa*-typing of all MRSA isolates yielded 24 *spa* types. t437, t899, t127, and t091 were the most predominant *spa* types, constituting 71.3% (78/108) of all of the isolates in this study. Other *spa* types, including t002, t034, t085, t114, t116, t163, t189, t377, t441, t528, t543, t571, t1751, t1764, t2874, t3485, t4549, t4792, t5554, and t9472, were distributed in fewer isolates (27.8%, 30/108). The MRSA isolates were also subjected to identified SCC*mec* types, and the types of 20 isolates could not be detected. The majority of MRSA isolates possessed SCC*mec* type IV, which was observed in 63.9% of isolates (69/108), including 50 for SCC*mec* type IVa, 17 for SCC*mec* type IVb and 2 for SCC*mec* type IVd. In addition, 9 isolates belonged to SCC*mec* III, and 10 isolates belonged to SCC*mec* V.

**Table 4:** The STs, *spa* types and SCC*mec* types of the MRSA strains isolated from retail food in China.

		No. (%) of positive isolates						
Method	Criterion	Total (*n* = 108)	Raw meat (*n* = 34)	Aquatic products (*n* = 31)	Quick-frozen meat (*n* = 20)	Ready-to-eat food (*n* = 11)	Edible mushroom (*n* = 7)	Vegetables (*n* = 5)
MLST	ST59	51(47.2)	14(41.2)	15(48.4)	5(25.0)	7(63.6)	7(100.0)	3(60.0)
	ST9	17(15.7)	7(20.6)	–	9(45.0)	1(9.1)	–	–
	ST1	9(8.3)	2(5.9)	4(12.9)	–	2(18.2)	–	1(20.0)
	ST398	7(6.5)	4(11.8)	–	3(15.0)	–	–	–
	ST7	5(4.6)	3(8.8)	1(3.2)	–	–	–	1(20.0)
	ST338	5(4.6)	–	4(12.9)	–	1(9.1)	–	–
	ST630	3(2.8)	1(2.9)	1(3.2)	1(5.0)	–	–	–
	ST188	2(1.9)	1(2.9)	1(3.2)	–	–	–	–
	ST3355	1(0.9)	1(2.9)	–	–	–	–	–
	ST943	1(0.9)	1(2.9)	–	–	–	–	–
	ST3304	1(0.9)	–	1(3.2)	–	–	–	–
	ST5	1(0.9)	–	–	1(5.0)	–	–	–
	ST6	1(0.9)	–	1(3.2)	–	–	–	–
	ST10	1(0.9)	–	–	1(5.0)	–	–	–
	ST45	1(0.9)	–	1(3.2)	–	–	–	–
	ST88	1(0.9)	–	1(3.2)	–	–	–	–
	ND^a^	1(0.9)	–	1(3.2)	–	–	–	–
spa	t437	48(44.4)	10(29.4)	18(58.1)	2(10.0)	8(72.7)	7(100.0)	3(60.0)
	t899	16(14.8)	7(20.6)	–	8(40.0)	1(9.1)	–	–
	t127	7(6.5)	1(2.9)	4(12.9)	1(5.0)	1(9.1)	–	–
	t091	6(5.6)	5(14.7)	1(3.2)	–	–	–	–
	t034	4(3.7)	1(2.9)	1(3.2)	2(10.0)	–	–	–
	t002	3(2.8)	1(2.9)	1(3.2)	1(5.0)	–	–	–
	t085	2(1.9)	–	1(3.2)	–	1(9.1)	–	–
	t114	2(1.9)	–	1(3.2)	–	–	–	1(20.0)
	t163	2(1.9)	2(5.9)	–	–	–	–	–
	t1751	2(1.9)	1(2.9)	–	1(5.0)	–	–	–
	t189	2(1.9)	1(2.9)	1(3.2)	–	–	–	–
	t441	2(1.9)	–	–	2(10.0)	–	–	–
	t377	2(1.9)	1(2.9)	–	1(5.0)	–	–	–
	t116	1(0.9)	–	1(3.2)	–	–	–	–
	t1764	1(0.9)	–	1(3.2)	–	–	–	–
	t2874	1(0.9)	–	–	–	–	–	1(20.0)
	t3485	1(0.9)	1(2.9)	–	–	–	–	–
	t4549	1(0.9)	–	–	1(5.0)	–	–	–
	t4792	1(0.9)	1(2.9)	–	–	–	–	–
	t5554	1(0.9)	–	1(3.2)	–	–	–	–
	t528	1(0.9)	–	–	1(5.0)	–	–	–
	t571	1(0.9)	1(2.9)	–	–	–	–	–
	t9472	1(0.9)	1(2.9)	–	–	–	–	–
SCC*mec*	Type III	9(8.3)	3(8.8)	5(16.1)	1(5.0)	–	–	–
	Type IVa	50(46.3)	10(29.4)	21(67.8)	5(25.0)	7(63.6)	4(57.1)	3(60.0)
	Type IVb	17(15.7)	7(20.6)	–	9(45.0)	1(9.1)	–	–
	Type IVd	2(1.9)	1(2.9)	–	–	–	–	1(20.0)
	Type V	10(9.3)	1(2.9)	2(6.5)	3(15.0)	1(9.1)	3(42.9)	–
	ND^a^	20(18.5)	12(35.3)	3(9.7)	3(15.0)	1(9.1)	–	1(20.0)

*^*a*^ND, not detected.*

**FIGURE 2:**
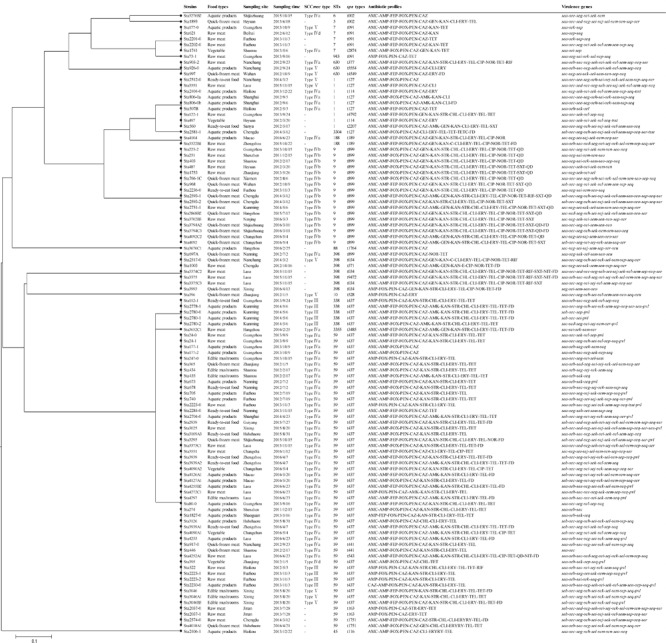
The UPGMA (unweighted pair group method with arithmetic mean) tree of the 7 multilocus sequence typing loci of MRSA isolates. This tree was generated using S.T.A.R.T (version 2).

A phylogenetic tree based on the 7 concatenated MLST sequences (Figure 2) shows the relatedness between the MRSA strains. Two different clusters were observed in this study (designated as A and B). Cluster A included ST1, ST3304, ST188, ST9, ST15, ST6, ST5, ST7, ST943, ST630, and ST88, and cluster B included ST398, ST10, ST338, ST3355, and ST59, which showed distant genetic relationships. STs correlated well with *spa* types, such as ST1-t127, ST188-t189, ST9-t899, ST7-t091, ST59-t437, and ST338-t437. Overall, the genetic diversity among MRSA isolates was higher based on different cities and different food sources. For different food products, more than three subtypes were found in each type of food, except edible mushrooms and vegetables. CC59-t437 (45.4%, 49/108) was the predominant clone in this study, but ST9-t899-SCC*mec* IVb was the predominant clone in quick-frozen meat and was found only in animal-derived food (raw meat, quick-frozen meat and ready-to-eat meat). In addition, 80% (4/5) of SCC*me*c III-ST338-t437 isolates were found in aquatic products from the city of Kunming. However, some strains showed correlations among geographical locations, such as Sta223-2 (isolated from Shenzhen), Sta251 (isolated from Guangzhou), Sta403 (isolated from Shantou), Sta487 (isolated from Heyuan), and Sta1753 (isolated from Zhanjiang), which were isolated from neighboring cities in this study and clustered into one subtype (ST9-t899-SCC*mec* IVb).

## Discussion

MRSA is a significant public health concern in humans and animals. The rate of mortality due to MRSA infections has remained high in recent years. In hospitals, the prevalence rates of MRSA in some Asian countries, such as Taiwan, China, Japan, and South Korea, can reach 70–80% (Chuang and Huang, 2013). For CA-MRSA, the prevalence varies substantially worldwide and ranges from less than 1% to more than 50% in different countries (Tristan et al., 2007; Deurenberg and Stobberingh, 2008). MRSA has also been isolated from animals, as reported in many previous studies (Leonard and Markey, 2008). It is important to identify the origin of food-related MRSA and to evaluate the potential pathogenicity of these MRSA isolates. From July 2011 to June 2016, 4300 retail food samples were collected from supermarkets, fairs and farmers’ markets, covering most of the provincial capitals of China. This wide-scale and systematic investigation of *S. aureus* from retail food in China supplements nationwide qualitative and quantitative data on the prevalence and levels of *S. aureus*. In this study, we determined the MRSA isolates from these food-related *S. aureus* isolates and found 108 MRSA isolates (6.83%) in various types of food products (raw meat, aquatic products, quick-frozen products, ready-to-eat food, edible mushrooms and vegetables) from most of the sampling cities (29/39, 74.4%) in China, which suggested that retail food in China could be contaminated with MRSA.

Many studies have evaluated the presence of MRSA in retail food. In China, among studies that sampled retail food, 6.07% of MRSA isolates were found in quick-frozen dumpling samples of Shaanxi province (Hao et al., 2015) and in 1.7% of chicken samples (Wang et al., 2013), whereas MRSA was present in 29.5% of grain products, meat products and dairy products in southwest China. Overall, these rates varied from our results in this study. This incidence may be attributed to a number of factors, such as the sample size, sampling site, types of samples or isolation methods. In 2015, (Wang et al., 2017) collected 1150 *S. aureus* isolates from retail markets from 203 cities in 24 provinces in China and found 91 isolates (7.9%) that were identified as MRSA by PCR. The MRSA isolates were distributed in raw meat, rice and flour products, vegetable salads, sandwiches, meat products, and eggs. Compared with other countries’ studies, the prevalence of MRSA from retail foods in China was not low. For instance, Ge et al. (2017) conducted a 1-year survey in 2010-2011 from 3520 retail meats in eight U.S. states and found that 1.9% of samples were positive for MRSA. An Italian survey found that 6 of 160 (3.75%) foods of animal origin harbored MRSA (Normanno et al., 2007). MRSA was present in 3.6% (15/421) of retail meat in Korea (Lee, 2003), 1.6% (5/318) of food animals in Spain (Lozano et al., 2009), 0.75% (20/2662) in Switzerland (Huber et al., 2010), 3% (11/367) in Greece (Papadopoulos et al., 2018), and 1.9% (2/103) in the United Kingdom (Hadjirin et al., 2015). Therefore, Chinese food safety regulators should improve hygiene and supervision efforts.

Currently, MRSA isolated from food-producing animals has been recognized as LA-MRSA. The worldwide emergence of LA-MRSA since 2005 has prompted many of the surveys to focus on retail meat as a potential vehicle for this new MRSA clone (Ge et al., 2017). In general, ST398 was recognized as the most typical type in LA-MRSA. ST398 in swine, cattle and other animal species has been analyzed in several publications (Walther et al., 2009; Fessler et al., 2010; Graveland et al., 2011). It reported that 24–100% of pig farmers, 37% of poultry farmers, 30–38% of cattle farmers and up to 45% of veterinarians are colonized with MRSA CC398 in the nares (Köck et al., 2014). In this study, most MRSA isolates (88.9%, 96/108) were isolated from animal-derived food (raw meat, aquatic products, quick-frozen products and ready-to-eat food), but only 7 MRSA isolates belonged to this ST type. STs of MRSA isolated from retail food focused on CC59 (ST59, ST338, and ST3355). This showed significant genetic uniformity with the predominant Asian CA-MRSA lineage, which can reach 35.8–76.7% with CA-MRSA in China (Yang et al., 2017). In general, the CC59 clone always carried SCC*mec* IV/V and was concentrated in *spa* t437 and t441. In 2013, Li et al. (2013) analyzed the 110 CC59 isolates from invasive and noninvasive diseases in China by MLST, SCC*mec* typing and *spa*-typing and found that 65.5% of the clones were ST59-t437-IVa. From February 2016 to January 2017 Yang et al. (2017), collected *S. aureus* strains in Beijing Children’s hospital from the respiratory tract, skin and soft tissue, and sterile sites in 104 child cases. Of these, 54.8% were categorized as community-associated SA (CA-SA) infections, and ST59-SCC*mec* IV-t437 (61.7%) was the most prevalent MRSA genotype. In addition to China, ST59 has also been reported in Vietnam, Japan, Australia and other countries with CA-MRSA infection (Tang et al., 2007; Coombs et al., 2010; Higuchi et al., 2010). In contrast, ST239 and ST5 were found in a nationwide study to be two major MRSA clones with unique geographic distributions in Chinese hospitals (Liu et al., 2009). Accordingly, these results implied that the major food-related MRSA in China was closer to CA-MRSA, a finding that should be brought to public attention.

In the present study, 15.7% (17/108) of isolates belonged to ST9, the secondary clones of which were found only in animal-derived food (raw meat, quick-frozen meat and ready-to-eat meat). According to previous studies, ST9 was the predominant *S. aureus* and MRSA genotype in pigs and related workers in Asia (Chuang and Huang, 2015). In 2008, ST9 MRSA was first found in Chinese pigs, and farm workers carried ST9-t899-SCC*mec* III-PVL-negative (Cui et al., 2009). Studies in Taiwan, Hong Kong, Malaysia and Thailand have since reported the prevalence of this type of LA-MRSA (Neela et al., 2009; Graveland et al., 2011; Larsen et al., 2012; Lo et al., 2012). ST9 is the most prevalent LA-MRSA in most Asian countries and differs from the European pig-associated clone (ST398) with regard to clonal type, SCC*mec* content and resistance profile (Ye et al., 2016). In China, ST9 strains always showed SCC*mec* III with *spa* t899. These characteristics were in accordance with our results, which showed that the ST9 MRSA isolated from our study showed ST9-t899-SCC*mec* III. Therefore, this portion of the food-related MRSA isolates was supposed to be LA-MRSA. However, it is worth noting that in this study, all ST9 MRSA isolates were resistant to more than 15 antibiotics and showed a broader range of antimicrobial resistance than ST59 MRSA (Figure 2). More than 80% of ST9 MRSA was resistant to erythromycin, ciprofloxacin, gentamicin, tetracycline and clindamycin. Currently, more evidence has implicated animals as reservoirs of antimicrobial-resistant bacteria and has indicated that animals can potentially transmit resistance genes to humans (Liu et al., 2018). Thus, more attention should be focused on this type of strain among food chains.

Except for CC59 and ST9, the remaining strains belonged to ST1 (8.4%, 9/107), ST398 (6.5%, 7/107), ST7 (4.7%, 5/107), ST630 (2.8%, 3/107), and ST188 (1.9%, 2/107). Of these strains, most STs correlated well with *spa* types, but seven ST398 isolates distributed in four different *spa* types. In addition, SCC*mec* types were not detected in most (Figure 2). This finding is consistent with the results of a previous study suggesting that divergent SCC*mec* elements were inserted into the (clonal) ST398 MSSA (van Duijkeren et al., 2008; Smith and Pearson, 2011). Therefore, further studies evaluating MSSA ST398 in retail food are needed to determine the reason for the correlation. In this study, ST1-t127 and ST7-t091 were also detected. These STs showed high genetic diversity among MRSA isolates based on different cities and different food sources, which is a common finding in isolates of human and animal origin (Franco et al., 2011; Hummerjohann et al., 2014). Thus, these types of *S. aureus* isolates have been relevant to a variety of clinical infections and have theoretical pathogenic potential.

As is usually observed with CA-MRSA strains, CA-MRSA ST59 isolates had significantly more pronounced virulence in various animal infection models than the geographically matched HA-MRSA clones ST5 and ST239 (Li et al., 2009, 2016). For the strains of ST59, the evolutionary acquisition of PVL, the higher expression of α-toxin and, possibly, the loss of a large portion of the β-haemolysin-converting prophage probably contribute to its higher pathogenic potential (Chen et al., 2013; Chen and Huang, 2014). In this study, we also investigated the PVL gene and found 24.07% PVL-positive MRSA isolates. All PVL-positive MRSA isolates belonged to CC59, including 22 ST59 isolates and four ST338 isolates. For LA-MRSA ST9 isolates, certain important virulence factors, such as PVL, are absent. Despite a lack of virulence factors, ST9 strains have been found to cause disease in humans (van Loo et al., 2007; Liu et al., 2009; Chuang and Huang, 2015). Therefore, the hazards of these strains for consumers cannot be ignored.

As an important foodborne pathogen, *S. aureus* is involved in most staphylococcal food poisoning (SFP) events due to staphylococcal enterotoxins, the virulence factors that are heat stable and proteolytic or demonstrate emetic activity (Grumann et al., 2014). In this study, we also investigated most of the enterotoxin genes of *S. aureus*. All food-related MRSA isolates harbored at least one of the SE genes. The percentages of MRSA isolates containing *sea, seb*, and *sec* all exceeded 50%, whereas *sed* and *see* were detected in only fourteen MRSA isolates (12.96%). Except for the *egc* cluster, which accounted for 29.4% (245/833) of the detected genes in the present study, other SE genes showed 48.4% (403/833) of the detected genes. Generally, SEA, followed by SED, is the enterotoxin most frequently associated with SFP, although outbreaks caused by SEB, SEC, and SEE have also been reported (Argudín et al., 2012). In contrast to classical SEs, the relationship between the novel SEs and SFP is not fully understood, but most of them (SEG, SEH and SEI, SER, SES, and SET) have been shown to be emetic after oral administration in a primate model (Argudín et al., 2010). In this study, 94 of 108 MRSA isolates (87.04%) harbored *egc* cluster genes (*seg, sei, sem, sen, seo*, and *seu*), and 94.4% (102/108) of isolates harbored one or more genes for other novel SEs or *tsst-1* genes. It is suggested that attention should not only be paid to classical enterotoxins but also to novel ones since an increasing number of foodborne outbreaks have been associated with novel enterotoxins.

Nowadays, multiple drug resistance is the most important characteristic of MRSA isolates. For the 26 clinically relevant antibiotics investigated, all MRSA isolates were resistant to more than three antibiotics. It seems higher than many previous studies in food-related MRSA isolates (Hanson et al., 2011; Shahraz et al., 2012; Ge et al., 2017; Tang et al., 2017). Interestingly, norfloxacin, clindamycin, trimethoprim/sulphamethoxazole 1:19, gentamycin and quinupristin/dalfopristin resistance were rare in MRSA isolated from non-animal sources (edible mushrooms and vegetables) (Supplementary Figure 2). The reason for this finding may be attributed to the food source. In general, most animal-derived food-related *S. aureus* came from animal farms that used these antibiotics as food supplements in animal feed (Wang et al., 2014), whereas *S. aureus* isolated from edible mushrooms or vegetables most likely originated in the environment. Furthermore, amikacin was the most commonly observed resistance antibiotic in aquatic product-related MRSA isolates, whereas gentamycin, quinupristin/dalfopristin, ciprofloxacin, and norfloxacin resistances were observed less frequently than other types of food isolates (Supplementary Figure 2). As we know, amikacin, gentamycin, kanamycin and streptomycin all belong to the aminoglycosides, which exert their bactericidal effects by irreversibly binding to the 30S ribosomal subunits of susceptible bacteria, inhibiting protein synthesis (Hammerberg et al., 1986). The most common clinical resistance mechanism to aminoglycosides is the structural modification of aminoglycosides by aminoglycoside-modifying enzymes (AMEs). Amikacin is a broad-spectrum semi-synthetic derivative of kanamycin and a poor substrate of many AMEs. Furthermore, even if amikacin is modified by AMEs, the modified amikacin can still bind to the 30S ribosomal subunit (Yuan et al., 2013). Therefore, amikacin is one of the most potent classes of antibiotics in *S. aureus* infection. In fact, the mechanism of MRSA resistance to amikacin is poorly understood. Thus, why amikacin resistance was higher in MRSA isolates from aquatic products should be further studied.

## Conclusion

Essential for human survival, food is the one of most basic necessities of life. In this study, we investigated food-related MRSA and determined its genetic background in China. MRSA isolates were found in most investigated cities and were observed in different types of food samples. ST59 was the predominant clone in food-related MRSA in China, which indicated that the major food-related MRSA isolates in China were closer to CA-MRSA. Moreover, as the major LA-MRSA in Asian populations, ST9 MRSA was the secondary clone and showed a broader range of antimicrobial resistance. Determination of staphylococcal toxin genes presented their virulence potential, and antimicrobial susceptibility testing further confirmed the severe situation of MRSA isolated in retail food in China. In addition, some antibiotics were also found to be higher in some types of food. However, further studies are required to determine the reason for this correlation and to elucidate the transmission routes of MRSA in relation to retail foods in order to provide the tools for preventing the spread of MRSA.

## Author Contributions

QW, JZ, SW, and TL conceived and designed the experiments. JH, FZ, and JD performed the experiments. SW, RP, and HZ analyzed the data. XW, LX, MC, and XY contributed reagents, materials, and analysis tools. SW and JW contributed to the writing of the manuscript.

## Conflict of Interest Statement

The authors declare that the research was conducted in the absence of any commercial or financial relationships that could be construed as a potential conflict of interest.

## Footnotes

1 http://spaserver2.ridom.de

2 https://pubmlst.org/saureus/
