# Supplementary material for: Prevalence and Characterization of Food-Related Methicillin-Resistant Staphylococcus aureus (MRSA) in China
Source: Front Microbiol. 2019 Feb 20;10:304. doi: 10.3389/fmicb.2019.00304 (PMC6391343; doi:10.3389/fmicb.2019.00304)
Supplement: Supplementary file 1 [file Data_Sheet_1.docx]

Supplementary Material

Prevalence and characterization of methicillin-resistant *Staphylococcus aureus* (MRSA) isolated from retail food in China

**Shi Wu #, Jiahui Huang #, Feng Zhang , Qingping Wu*, Jumei Zhang, Rui Pang, Haiyan Zeng, Xiaojuan Yang, Moutong Chen, Juan Wang, Jingsha Dai, Liang Xue, Tao Lei, Xianhu Wei**

*** Dr. Qingping Wu:** [**wuqp203@163.com**](mailto:wuqp203@163.com)

# Supplementary Figures and Tables

## Supplementary Figures


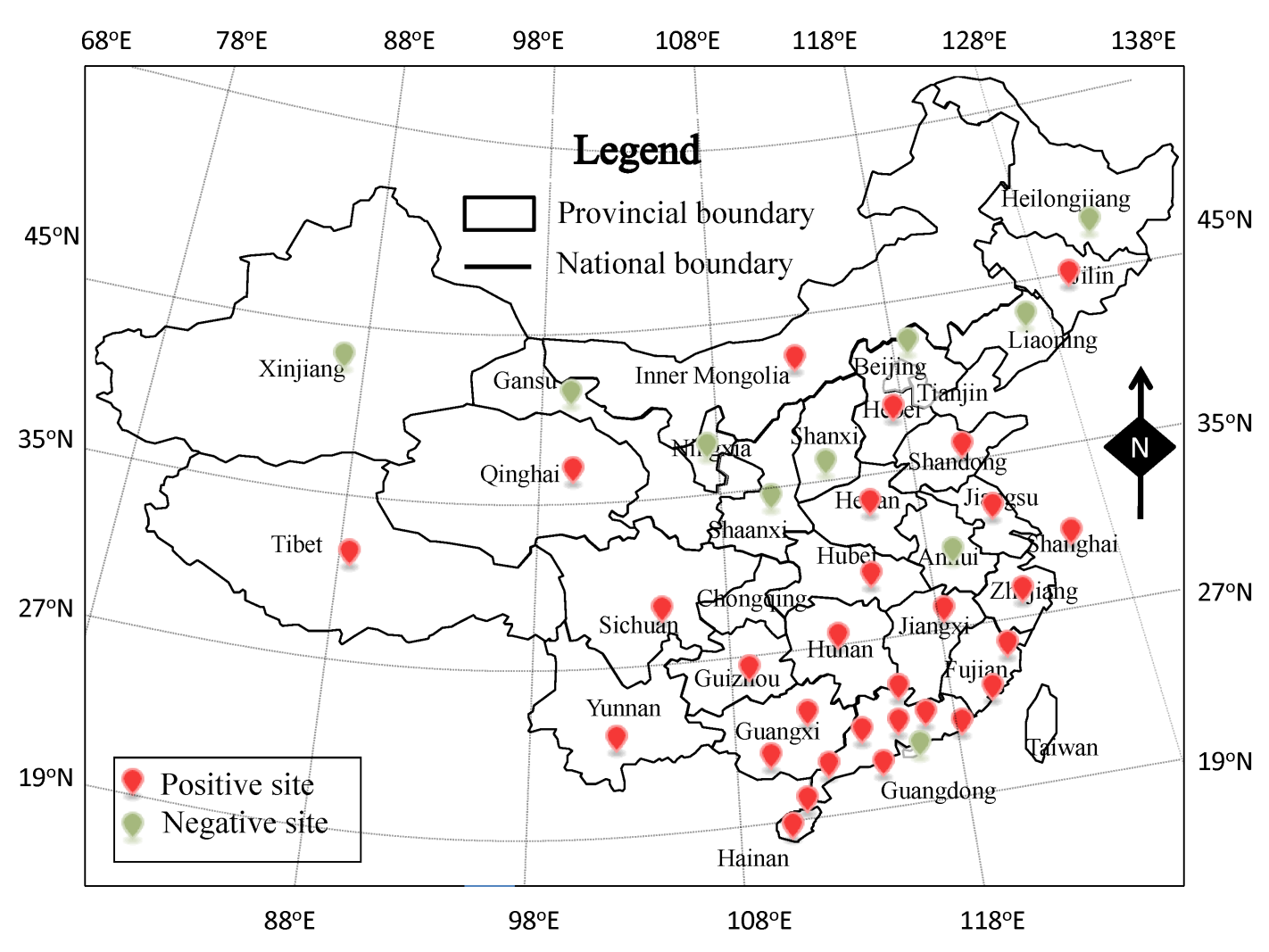


**Supplementary Figure 1.** The locations of the sampling sites for this study in China.


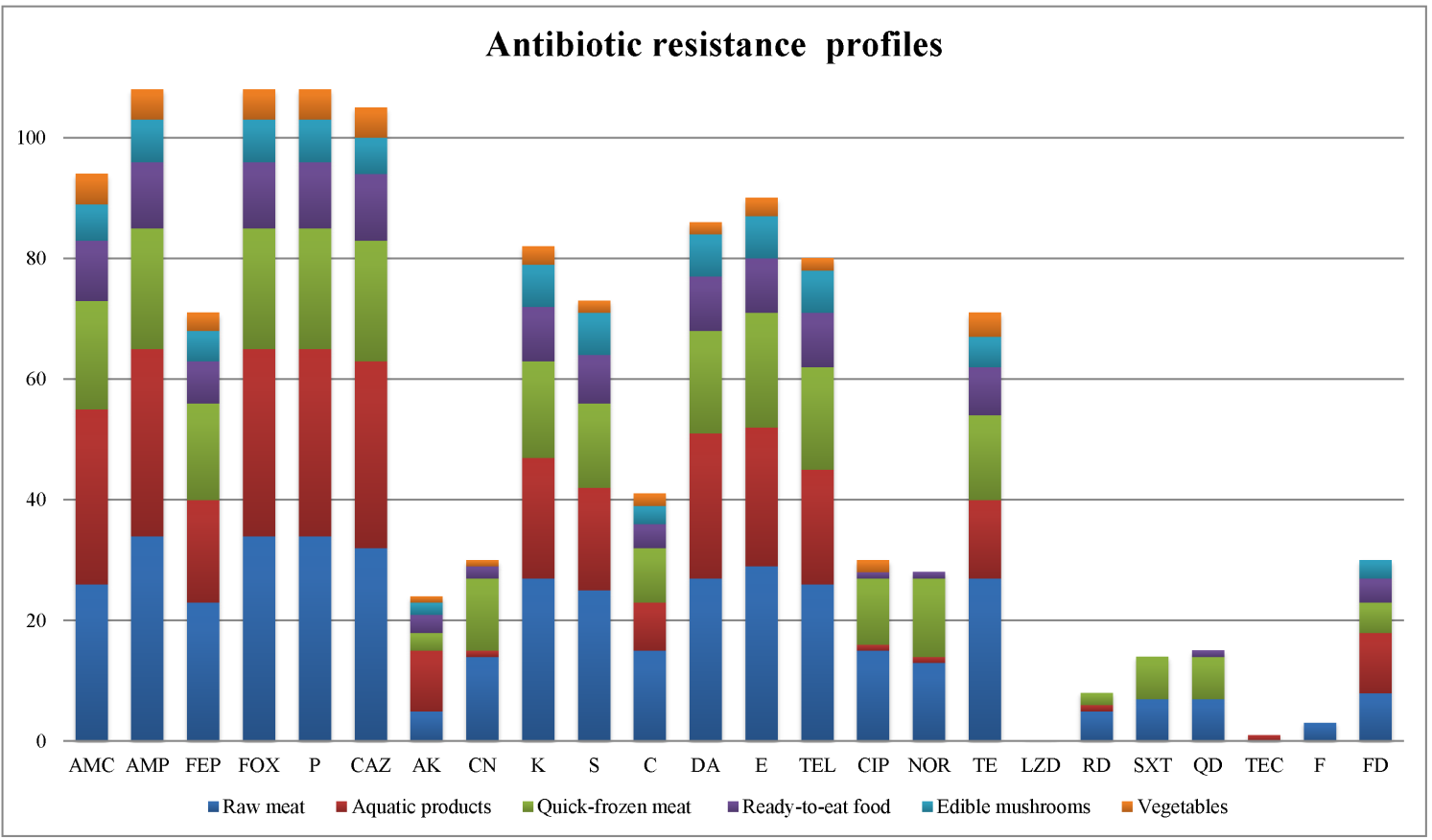


**Supplementary Figure 2.** The antibiotic resistance profiles of MRSA isolates in different food types

## Supplementary Tables

**Supplementary Table 1.** Sequences of primers used for detection of virulence genes in this study

| Gene target | Primer sequences (5’-3’) | Product sizes(bp) | References |
| --- | --- | --- | --- |
| *pvl* | F: ATCATTAGGTAAAATGTCTGGACATGATCCA | 433bp | X. Wang et al. (2012) |
|  | R: GCATCAASTGTATTGGATAGCAAAAGC |  |  |
| *tsst* | F: AAG CCC TTT GTT GCT TGC G | 447bp | Avanish K. Varshney et al. (2009) |
|  | R:ATC GAA CTT TGG CCC ATA CTT T |  |  |
| *sea* | F: GAAAAAAGTCTGAATTGCAGGGAACA | 561bp | I. Ote et al.Veterinary Microbiology (2011) |
|  | R: CAAATAAATCGTAATTAACCGAAGGTTC |  |  |
| *seb* | TCGCATCAAACTGACAAACG | 477bp | Avanish K. Varshney et al. (2009) |
|  | GCAGGTACTCTATAAGTGCCTGC |  |  |
| *sec* | F: AGATTTAGCAAAGAAGTACAAAGATG | 490bp | Avanish K. Varshney et al. (2009) |
|  | R: AAGGTGGACTTCTATCTTCACACTT |  |  |
| *sed* | F: CTA GTT TGG TAA TAT CTC CT | 319bp | I. Ote et al.Veterinary Microbiology (2011) |
|  | R: TA ATG CTA TAT CTT ATA GGG |  |  |
| *see* | F: ACCGATTGACCGAAGAAAAA | 264bp | Avanish K. Varshney et al. (2009) |
|  | R: ATTGCCCTTGAGCATCAAAC |  |  |
| *seg* | F: AGAATTAGCTAACAATTATAAAGATAAAAAAG | 496bp | Avanish K. Varshney et al. (2009) |
|  | R: TCAGTGAGTATTAAGAAATACTTCCAT |  |  |
| *she* | F: TGATTTAGCTCAGAAGTTTAAAAATAAAAATG | 466bp | Avanish K. Varshney et al. (2009) |
|  | R: TTTCTTAGTATATAGATTTACATCAATATG |  |  |
| *sei* | F: TGGAACAGGACAAGCTGAAA | 529bp | Avanish K. Varshney et al. (2009) |
|  | R: TGTTTGCCATTAACCCAAAG |  |  |
| *sej* | F: ATGAAAAAAACAATATTTATACTGATTTTCTCCC | 807bp | Avanish K. Varshney et al. (2009) |
|  | R: TCTACAGAACCAAAGGTAGACTTATTAATAC |  |  |
| *sek* | F: ATGAATCTTATGATTTAATTTCAGAATCAA | 545bp | Avanish K. Varshney et al. (2009) |
|  | R: ATTTATATCGTTTCTTTATAAGAAATATCG |  |  |
| *sel* | F: ATGAAAAAAAGATTATTATTTGTAATTGTTATTAC | 723bp | Avanish K. Varshney et al. (2009) |
|  | R: ATCATCTTTTTGAAATTTCGACATCTAG |  |  |
| *sem* | F: ATGAAAAGAATACTTATCATTGTTGTTTTATTG | 720bp | Avanish K. Varshney et al. (2009) |
|  | R: CTTCAACTTTCGTCCTTATAAGATATTTC |  |  |
| *sen* | F: ATAAAAAATATTAAAAAGCTTATGAGATTGTTC | 777bp | Avanish K. Varshney et al. (2009) |
|  | R: ACTTAATCTTTATATAAAAATACATCAATATG |  |  |
| *seo* | F: TATGTAGTGTAAACAATGCATATGCA | 685bp | Avanish K. Varshney et al. (2009) |
|  | R: TCTATTGTTTTATTATCATTATAAATTTGCAAAT |  |  |
| *sep* | F: TTAGACAAACCTATTATCATAATGGAAGT | 618bp | Avanish K. Varshney et al. (2009) |
|  | R: TATATAAATATATATCAATATGCATATTTTTAGACT |  |  |
| *seq* | F: GGAAAATACACTTTATATTCACAGTTTCA | 539bp | Avanish K. Varshney et al. (2009) |
|  | R: ATTTATTCAGTTTTCTCATATGAAATCTC |  |  |
| *ser* | F: AGCGGTAATAGCAGAAAATG | 363bp | Avanish K. Varshney et al. (2009) |
|  | R: TCTTGTACCGTAACCGTTTT |  |  |
| *seu* | F: AATGGCTCTAAAATTGATGG | 215bp | Avanish K. Varshney et al. (2009) |
|  | R: ATTTGATTTCCATCATGCTC |  |  |
